# Sclerostin Inhibition in the Management of Osteoporosis

**DOI:** 10.1007/s00223-016-0126-6

**Published:** 2016-03-26

**Authors:** Natasha M. Appelman-Dijkstra, Socrates E. Papapoulos

**Affiliations:** Center for Bone Quality, Leiden University Medical Center, Albinusdreef 2, 2333 ZA Leiden, The Netherlands

**Keywords:** Osteoporosis, Sclerostin, Bone modeling, Bone remodeling, Blosozumab, Romosozumab

## Abstract

The recognition of the importance of the Wnt-signaling pathway in bone metabolism and studies of patients with rare skeletal disorders characterized by high bone mass identified sclerostin as target for the development of new therapeutics for osteoporosis. Findings in animals and humans with sclerostin deficiency as well as results of preclinical and early clinical studies with sclerostin inhibitors demonstrated a new treatment paradigm with a bone building agent for the management of patients with osteoporosis, the antifracture efficacy, and long-term tolerability of which remain to be established in on-going phase III clinical studies. In this article we review the currently available preclinical and clinical evidence supporting the use of sclerostin inhibitors in osteoporosis.

## Introduction

Available pharmacological agents for the treatment of osteoporosis reduce the risk of fractures but cannot restore the low mass and the deteriorated architecture of the skeleton of patients with severe disease. These agents reduce bone fragility by correcting the imbalance between bone resorption and bone formation by either decreasing bone resorption (e.g., bisphosphonates, denosumab) or stimulating bone formation (e.g., PTH peptides, PTHrP analogs) by different molecular mechanisms of action. Reduction of bone resorption, though essential for the maintenance or improvement of bone strength, cannot replace already lost bone. For this, specific stimulation of bone formation is required. Thus, in theory, optimal pharmacological management of osteoporosis should aim at decreasing bone resorption and stimulating bone formation at all skeletal envelopes. Such approach will not only prevent the structural decay of bone tissue but will also substantially increase bone mass leading to enhanced reduction of the risk of fractures particularly at sites with predominant cortical bone.

A mechanistic study examined this hypothesis and tested the effect of daily injections of teriparatide together with 6-monthly injections of denosumab in women with postmenopausal osteoporosis [1]. This combination, that allows continuous stimulation of bone formation by teriparatide by blocking its concurrent stimulating effect on bone resorption by denosumab, increased spine and hip BMD to levels significantly higher than either monotherapy after 1 year. While the study, by design, did not provide any evidence of improved antifracture efficacy of the combination therapy, results obtained with High-Resolution pQCT of the distal tibia suggested that it may have a better effect than either teriparatide or denosumab on the biomechanical competence of bone [2]. The discovery of the importance of the Wnt-signaling pathway in bone metabolism [reviewed in 3] and studies of patients with rare skeletal disorders characterized by high bone mass identified sclerostin—a natural inhibitor of the Wnt pathway that reduces bone formation—as a target for the development of new therapeutics that may fulfill the requirements for improved treatment for osteoporosis [4–6]. In this article we review the currently available preclinical and clinical evidence supporting the use of sclerostin inhibitors in the management of patients with osteoporosis.

### Sclerostin Deficiency

**Fig. 1:**
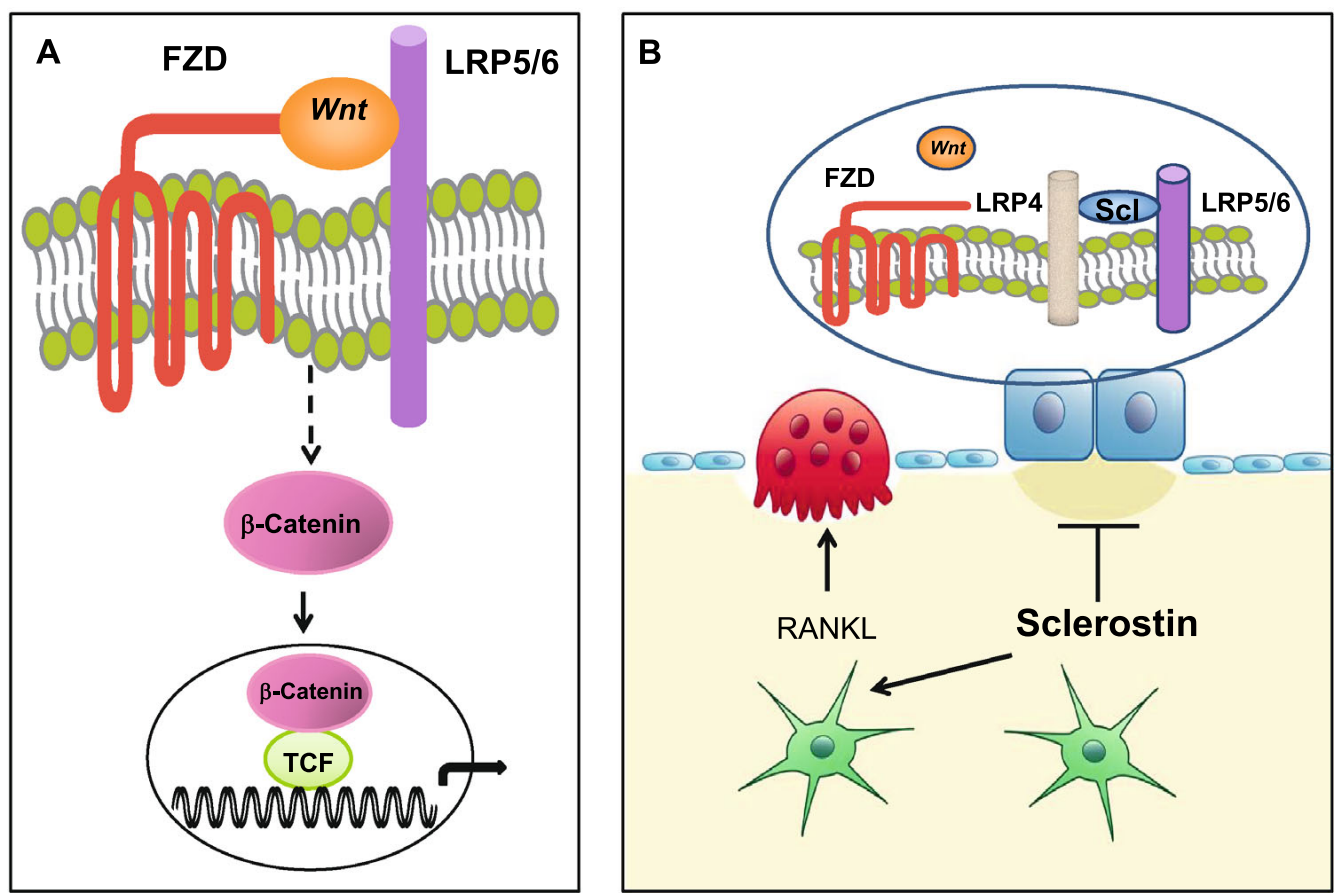
Schematic presentation of the canonical Wnt-signaling pathway and of the effect of sclerostin on bone cells. **a** Wnts bind to the receptor complex of frizzled (FZD) and LRP5/6, prevent the degradation of beta-catenin, and increase its accumulation in the cytoplasm; beta-catenin is translocated to the nucleus where it associates with transcription factors to control transcription of target genes in osteoblasts. **b** Osteocyte-produced sclerostin is transported to the bone surface and acts on osteoblasts to reduce bone formation by disabling the association of Wnts with their co-receptors and inhibiting the Wnt pathway in osteoblasts, an action facilitated by LRP4; sclerostin also stimulates the production of RANKL by neighboring osteocytes and osteoclastic bone resorption

Sclerosteosis and van Buchem disease are two rare, autosomal recessive, sclerosing bone disorders characterized by high bone mass and increased bone strength caused by defects of the *SOST* gene in chromosome 17q12-21 that encodes sclerostin [7–12]. While sclerosteosis is caused by inactivating mutations of *SOST,* a 52 kb homozygous noncoding deletion 35 kb downstream of the *SOST* gene containing a regulatory element for *SOST* transcription is the cause of van Buchem disease. These defects lead to impaired synthesis of sclerostin, a secreted glycoprotein with sequence similar to the DAN (differential screening-selected gene aberrative in neuroblastoma) family of proteins. Sclerostin is secreted by mature osteocytes embedded in the mineralized matrix and inhibits bone formation at the bone surface by binding to LRP5/6 co-receptors and thereby antagonizing canonical, beta-catenin dependent, Wnt signaling in osteoblasts [13–17]. Sclerostin binds to the first propeller of the LRP5/6 receptor and disables the formation of complexes of Wnts with frizzled receptors and the co-receptors LRP5/6, an action facilitated by the LRP4 receptor [18–20] (Fig. 1). Moreover, sclerostin acts on neighboring osteocytes and increases RANKL expression and the RANKL/OPG ratio and thereby stimulates osteoclastic bone resorption having, thus, a catabolic effect in bone in addition to its negative effect on bone formation [21, 22]. The clinical, biochemical, and radiological features of sclerosteosis and van Buchem disease have been described in detail [23–31] and we will further discuss only features of these diseases that may assist in the interpretation of results obtained in preclinical and clinical studies of sclerostin inhibition.

Targeted deletion of the *SOST* gene in mice greatly increased mineral density of vertebrae and whole leg, as well as the volume and strength of both trabecular and cortical bone [32]. MicroCT analysis showed, in addition, significant increases in the thickness of the distal femur and of the cortical area of the femur shaft due to increased rates of bone formation, assessed by histomorphometry, at trabecular and cortical (endosteal and periosteal) compartments while osteoclast surface was not different from that of wild-type animals; for example, compared with wild-type female mice, mineralizing surfaces, mineral apposition rate, and bone formation rate of the periosteal surface of cortical bone of *SOST*-KO mice increased by 249, 143, and 396 %, respectively. Bone had normal lamellar structure and, similar to humans with sclerosteosis, the increased mineral density was not associated with increased bone matrix mineralization [33]. These findings are clearly different from those observed in animal models of osteopetrosis with high mineral density. As in patients with sclerosteosis and van Buchem disease, serum calcium and phosphate concentrations in *SOST*-KO mice were not different from those of their wild-type littermates while serum osteocalcin values were increased with no changes in serum TRAP5b values. Follow-up of these mice showed that BMD increased progressively from 1 to 4 months of age and at a slower rate thereafter reaching a peak that was maintained up to 18 months [4]. It should be noted that in patients with sclerosteosis and van Buchem disease the disease stabilizes after the 3^rd^ decade of life and no new complications resulting from continuing bone overgrowth are generally observed. Importantly, *SOST*-KO mice had no apparent extraskeletal abnormalities.

The restricted expression of sclerostin in bone and the lack of complications in organs other than the skeleton in humans and animals with sclerostin deficiency made this protein an attractive target for the development of a novel anabolic therapy for osteoporosis. This notion was further supported by findings in human heterozygous carriers of sclerosteosis who have decreased serum sclerostin levels associated with high normal or increased BMD and increased bone strength without any clinical signs or complications of the disease [27, 28] indicating that sclerostin production can be reduced without any adverse skeletal effects.

### Preclinical Studies with Sclerostin Inhibitors

To assess the effects of sclerostin inhibition on bone metabolism and strength neutralizing antibodies to sclerostin (Scl-Ab) were administered to different animal models for varying periods of time (Table 1). In an early study, Scl-Ab was given subcutaneously twice-weekly for 5 weeks to rats ovariectomized (OVX) at the age of 6 months and left untreated for 1 year [34]. Treatment of this rodent model of osteoporosis was associated with dramatic increases in bone mass and improvement in bone strength at all skeletal sites examined. Remarkably, this short-term treatment with Scl-Ab not only completely reversed OVX–induced bone loss, but increased bone mass and strength to levels greater than those of sham-operated control animals. Histologically, bone formation increased markedly in trabecular, endocortical, and periostal surfaces leading to increases in trabecular and cortical thickness and reduction of cortical porosity. Increases in both mineral apposition rate and mineralizing surfaces suggested that short exposure to sclerostin inhibitor increases both the activity and the number of osteoblasts. The anabolic effect of Scl-Ab in rodents did not appear to depend on the prevalent rate of bone turnover and was not affected by pretreatment or co-treatment with alendronate of OVX rats with osteopenia [35]. Differently from PTH/PTHrP peptides, the high bone-forming activity of Scl-Ab was not associated with an increase in bone resorption. Instead a decrease of osteoclast surface was observed, suggesting a functional uncoupling between bone formation and bone resorption, as also observed in the studies of the *SOST*-KO mice. The effect of Scl-Ab on bone formation was reversible upon discontinuation of treatment. Similar results on both trabecular and cortical bone mass were generally observed in other rodent models treated with Scl-Ab (e.g., 10-month-old intact female rats immediately after OVX, OVX rats with established osteopenia, aged male rats, orchidectomized male rats, mouse models of immobilization, glucocorticoid-treated mice, and mice models of osteogenesis imperfecta) [4, 36–43]. Treatment with Scl-Ab was also reported to increase bone formation, mass and strength at the site of fracture in animal models of fracture healing [4, 44–46], and to completely reverse the bone loss and the deterioration of several bone mechanical and microstructural properties in a mouse model of chronic colitis [47]. Finally, despite marked increases in bone volume with Scl-Ab, matrix mineralization was not affected indicating that treatment does not negatively impact bone matrix quality [48].

**Table 1:** Biomechanical competence of bones (strength) of animals treated with sclerostin antibody

Animal	Age	Treatment	Duration	Strength	Ref.
Intact gonads					
Rats (M)	6 month	Scl-AbVI 2/week	9 week	↑F, V(nd)	[64]
Rats (M)	7 month	Scl-AbIII 2/week	7 week	↑V/F	[46]
Rats (M)	16 month	Scl-AbII 2/week	5 week	↑V/F	[37]
Cynos (Fe)	3–5 years	Scl-AbIV 1/month	2 week	↑V↔F	[49]
Cynos (M)	4–5 years	Scl-AbV 2/week	10 week	↑V/F	[46]
Estrogen deficiency (OVX)					
Rats	18 month^a^	Scl-AbII 2/week	5 week	↑V/F	[34]
Rats	6 month^b^	Scl-AbVI 1/wh	26 week	↑V/F	[50]
Rats	6.5 month^c^	Scl-A III 1/week	6 week	↑V, F(nd)	[35]
Cynos	≥9 years	ROMO 1/week^d^	12 month	↑V/F	[65]

*Cynos* cynomolgus monkeys, *Scl-Ab* sclerostin antibody, *ROMO* romosozumab, *V* vertebra, *F* femur, *nd* not examined

^a^OVX at 6 months

^b^OVX at 4 months

^c^OVX at 3.5 month

^d^Start treatment 4 month after OVX

Treatment of intact female cynomolgus monkeys with two once-monthly subcutaneous injections of different doses of Scl-Ab induced dose-dependent increases in bone formation on trabecular, periosteal, endocortical, and intracortical surfaces associated with significant gains in BMC/BMD [49]. Serum P1NP levels peaked 2 weeks after the first injection and 1 week after the second injection returning to baseline at the end of the treatment interval. There was no clear effect of Scl-Ab treatment on the bone resorption marker serum CTX. Biomechanical testing demonstrated a highly significant increase in the strength of vertebrae of animals treated with two injections of Scl-Ab compared with vehicle-treated animals while bone strength of the femoral diaphysis increased but not significantly. At both sites strong correlations between bone mass and bone strength were observed indicating that the changes in bone strength were due to the induced increases in bone mass. Thus, short-term exposure of different animal models to Scl-Ab was associated with remarkable changes of bone homeostasis, mass, and strength. Such changes occurred at all skeletal compartments and demonstrated that bone formation and resorption can be modulated in opposite directions by an inhibitor of sclerostin.

Two studies provided insight into the long-term use and the mechanism of action of Scl-Ab on bone metabolism. The first study, examined the effect of weekly injections of Scl-Ab given to 6-month-old OVX rats with osteopenia for 26 weeks. BMD of the spine and the tibia increased progressively through 26 weeks of treatment and was associated with increases in trabecular and cortical bone mass and strength at multiple skeletal sites [50]. Lumbar trabecular and endocortical and periosteal bone formation rates increased and peaked at 6 weeks of treatment with a gradual decline thereafter while osteoclast surface and eroded surface were significantly lower in Scl-Ab-treated OVX animals than in controls at all time points. These important observations reveal that while the early gains of bone mass with Scl-Ab treatment are due to the strong stimulation of bone formation combined with reduced osteoclastic activity, later gains may be attributed to persistently lower osteoclast activity and closure of the remodeling space combined with residual stimulation of osteoblasts at trabecular and endocortical surfaces. This study provides important, yet intriguing, information about the long-term effects of treatment with sclerostin inhibitors and raises questions about potential bone-site specificity of treatment in the long term as well as of optimal duration of treatment of humans with osteoporosis with Scl-Ab.

**Fig. 2:**
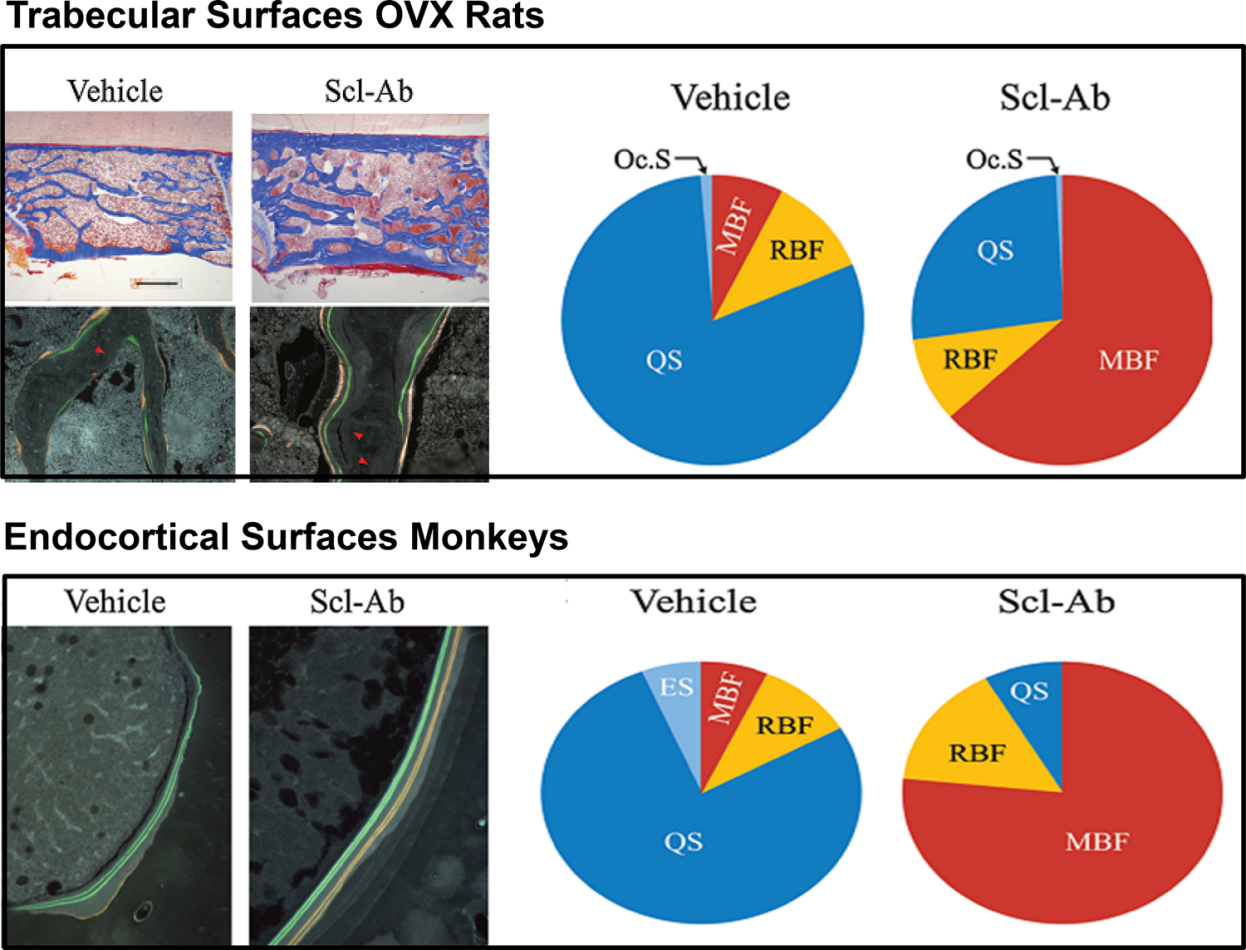
*Upper panel* Trabecular surfaces (L2) of OVX rats treated with vehicle or Scl-Ab. Surfaces were characterized as modeling-based bone formation (MBF), remodeling-based bone formation (RBF), quiescent (QS) or osteoclastic (OCs), and expressed as % of the total surface. *Lower panel* Endocortical surfaces (proximal diaphysis) of male cynomolgus monkeys. Bone surfaces are characterized as modeling-based bone formation (MBF), remodeling-based bone formation (RBF), quiescent (QS) or eroded surfaces (ES), and expressed as  % of the total surface. (From Ref. [51])

**Fig. 3:**
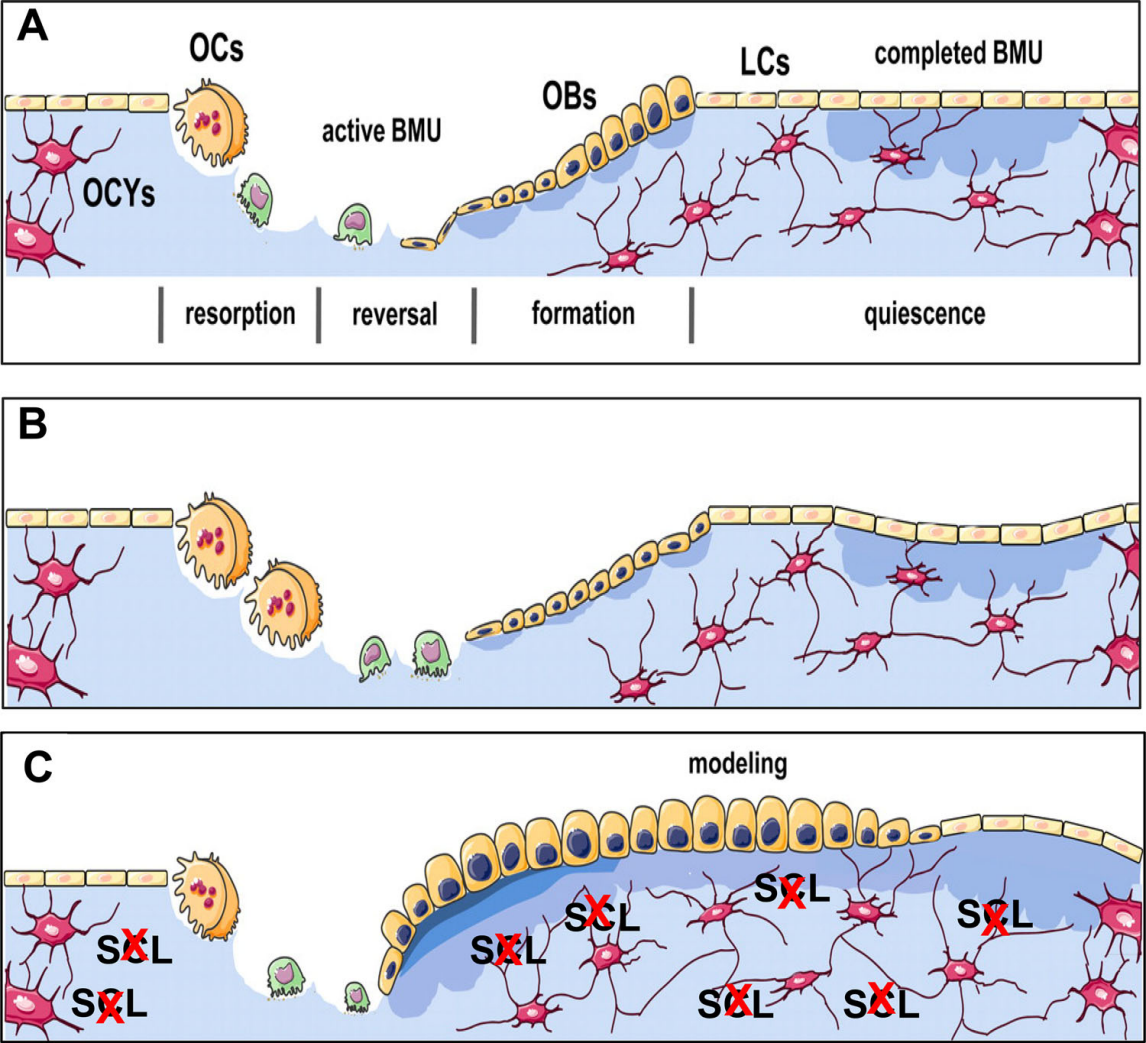
Bone remodeling and modeling under physiological conditions, in osteoporosis, and during treatment with sclerostin inhibitors. **a** Within an active BMU bone is constantly removed by osteoclasts (OCs) and new bone matrix is produced by osteoblasts (OBs), at sites where bone resorption has occurred with the amount of bone formed being equal to the amount of bone resorbed. Once the BMU is completed, osteoblasts become entrapped as osteocytes (OCYs) into the newly formed matrix, remain on the bone surface as lining cells (LCs), or undergo apoptosis. Bone then remains in the quiescent phase until a new BMU is initiated. **b** In osteoporosis, bone resorption is increased and bone formation is decreased, resulting in a loss of bone. **c** Inhibition of osteocyte-produced sclerostin decreases bone resorption but mostly increases both remodeling-based and modeling-based bone formation, thereby causing a striking increase in bone formation, particularly in areas that were not previously resorbed (modeling). (Modified from original Fig. 1 of Ref. [52])

To further characterize the specific effects of Scl-Ab on bone metabolism, the second study examined bone biopsies from OVX rats and intact male cynomolgus monkeys treated with Scl-Ab for 5 and 10 weeks, respectively [51]. Results showed that the majority of new bone formation was modeling based, occurring at quiescent surfaces (Fig. 2), and was associated with constant reduction of bone resorption. Treatment increased the rate of activation of new modeling surfaces while it decreased the rate of activation of bone remodeling surfaces and extended the formation period of existing remodeling sites. These observations support the notion of a new treatment paradigm for osteoporosis in line with the theoretical considerations discussed in Introduction and compatible with a true anabolic response ([52]; Fig. 3). We have previously suggested that agents that stimulate bone formation should be distinguished into bone forming (e.g., teriparatide) and anabolic [53]. The results of this study illustrate the differences in mechanism of action between the two classes of bone formation-stimulating agents at the bone tissue level, the clinical relevance of which remains to be established in the on-going human studies.

### Clinical Studies with Sclerostin Inhibitors

Information about three sclerostin inhibitors, all monoclonal humanized neutralizing antibodies, are currently in the public domain [romosozumab or AMG 785 (Amgen and UCB), blosozumab (Elli Lilly), and BPS804 (Novartis)].

The first human, placebo-controlled, study of 72 healthy men and postmenopausal women, demonstrated a dissociation of bone turnover marker responses following single subcutaneous or intravenous injections of romosozumab [54]. With the highest dose used (10 mg/kg sc), the bone formation markers serum P1NP, BAP, and osteocalcin increased rapidly and progressively reaching peaks of 184, 126, and 176 % of baseline values, respectively, after about 30 days and returned to baseline after about 2 months. In contrast, the bone resorption marker, serum CTX, decreased by a maximum 54 % of baseline values about 14 days after the antibody injection and returned to baseline after 2 months. This early response to a single injection of romosozumab is in agreement with the uncoupling of osteoblast and osteoclast activities observed in the animal studies. BMD of lumbar spine and total hip increased significantly by 5.3 and 2.8 %, respectively, on day 85. The pharmacodynamic response of different doses of romosozumab, up to three sc injections, given once-monthly to postmenopausal women and men with low bone mass was consistent with the results of the single-dose study showing early increases in bone formation markers and decreases in serum CTX [55]. In a subset of subjects of this study, changes of vertebral trabecular and cortical bone were examined by QCT and HR-QCT of the spine at 3 and 6 months, respectively. Compared with placebo, romosozumab treatment resulted in rapid improvements in trabecular and cortical bone mass and structure as well as of whole bone stiffness. These gains were maintained or improved in the 3 months following administration of the last dose of romosozumab. Improvements were also observed in microstructural aspects of both trabecular and cortical bone [56].

In a study of similar design, single or multiple doses of blosozumab given either subcutaneously or intravenously for up to 8 weeks to postmenopausal women, aged between 40 and 80 years, increased lumbar spine BMD dose-dependently, up to 7.7 % after 85 days; total hip BMD did not change significantly after either single or multiple doses of blosozumab [57]. There were dose-dependent changes of bone turnover markers of similar magnitude and direction as those observed with romosozumab. No serious adverse effects were observed with either sclerostin inhibitor.

**Fig. 4:**
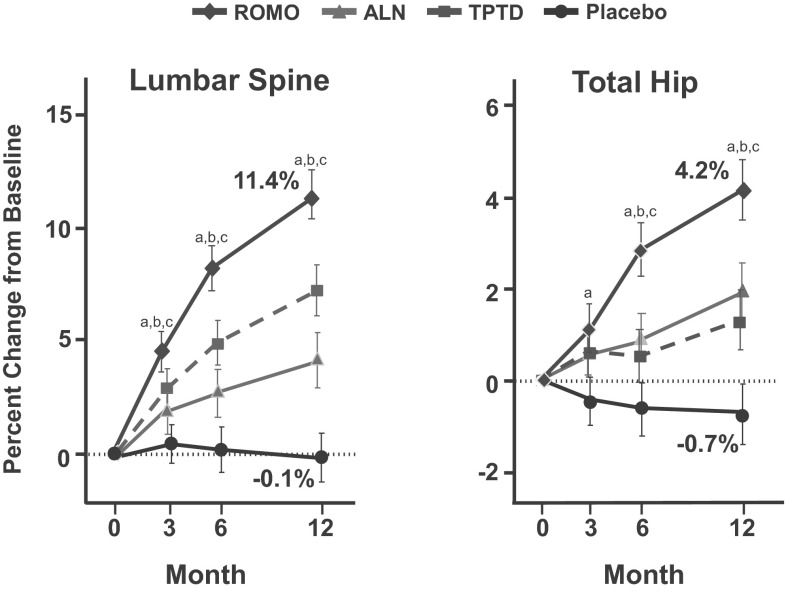
Percent changes of lumbar spine and total hip BMD during treatment of postmenopausal women with low bone mass with romosozumab (ROMO) 210 mg once-monthly sc, teriparatide (TPTD) 20 μg daily sc, alendronate (ALN) 70 mg once-weekly orally, or placebo. *a* = *p* < 0.05 between ROMO and placebo, *b* = *p* < 0.02 between ROMO and ALN, *c* = *p* < 0.02 between ROMO and TPTD (From Ref. [58])

**Fig. 5:**
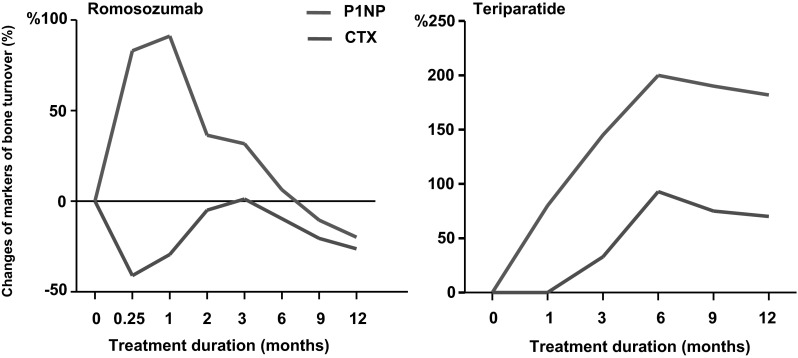
Schematic presentation of changes in the levels of serum biochemical markers of bone formation (P1NP) and bone resorption (CTX) during treatment with subcutaneous injections of either romosozumab 210 mg once-monthly or teriparatide 20 μg daily for 1 year. (From Ref. [59]; original data for romosozumab from Ref. [58] and for teriparatide from Ref. [66])

The efficacy and tolerability of romosozumab was examined in a phase II study of 419 postmenopausal women, aged 55–85 years with BMD T-scores between <−2.0 and −3.5 [58]. In this study, different doses and dosing intervals of subcutaneous injections of romosozumab were compared with placebo, oral alendronate 70 mg weekly, and subcutaneous teriparatide 20 μg daily. All women received calcium and vitamin D supplements and were randomly assigned to receive subcutaneous injections of placebo or romosozumab either once-monthly (70, 140, 210 mg) or once every 3 months (140, 210 mg). The primary efficacy point of the study was the change of spine BMD after 12 months. Three hundred and eighty-three (91 %) participants completed the study protocol. All doses of romosozumab induced significant gains in BMD. The highest dose of romosozumab used, 210 mg once-monthly, increased BMD at the spine (11.3 %), total hip (4.1 %), and femoral neck (3.7 %) after 1 year. These increases were significantly higher than those observed in women treated with either alendronate or teriparatide (Fig. 4). No statistically significant differences in BMD of the distal radius were observed between any of the three treatment groups and placebo. Markers of bone formation increased 1 week after the initial injection of romosozumab and reached a peak after 1 month. Thereafter, they decreased progressively returning to baseline values between 2 and 9 months of treatment reaching values significantly lower than baseline at 12 months. As in the phase I study, serum CTX levels decreased early after the first injection to a nadir of about 50 % of baseline after 15 days returning to baseline at 3 months and decreasing again significantly to −26 % of baseline at 12 months. These results illustrate that the action of sclerostin inhibitor on bone turnover is different from that of teriparatide as was already suggested in the animal and phase I human studies as well as in the observed changes of bone turnover markers in other clinical studies with teriparatide ([59]; Fig. 5). The incidence of adverse events was similar among all groups of studied women with the exception of mild reactions at the injection sites of romosozumab. One patient treated with romosozumab was diagnosed with breast cancer during the trial that was not considered to be treatment related. Antibodies with in vitro neutralizing activity were detected in 3 % of patients on romosozumab that had no effect on treatment outcomes. Continuation of romosozumab treatment 210 mg once-monthly for a second year increased further spine and total hip BMD to total gains of 15.2 and 5.5 %, respectively. The slope of this increase was, however, different from that during the first year of treatment [60]. Women were then randomized to receive denosumab or placebo for an additional year. Women who transitioned to denosumab continued to accrue BMD at a rate similar to that with romosozumab during the second year to a total of 19.4 % at the spine and 7.1 % at the total hip, while in those who transitioned to placebo BMD returned towards pretreatment levels. Interestingly, during the second year of romosozumab treatment serum levels of both P1NP and CTX remained below baseline indicating continuous stable decrease of bone turnover with prolongation of treatment. In patients who were switched to denosumab bone turnover markers decreased further to levels previously described with this antibody while in those who discontinued treatment serum P1NP levels gradually returned to pretreatment values and serum CTX levels, after an initial increase above baseline, gradually returned towards pretreatment values [60]. The pattern of changes of serum CTX values following romosozumab discontinuation was similar to that observed after discontinuation of denosumab [61]. Although the magnitude of these changes was different, due to the different degrees of suppression of bone resorption by the two treatments, levels of peak increases above baseline values were very similar illustrating that romosozumab has a genuine antiresorptive action and support the notion that this is exerted by a RANKL-dependent mechanism. Phase III clinical studies with fracture endpoints are currently performed with romosozumab.

**Fig. 6:**
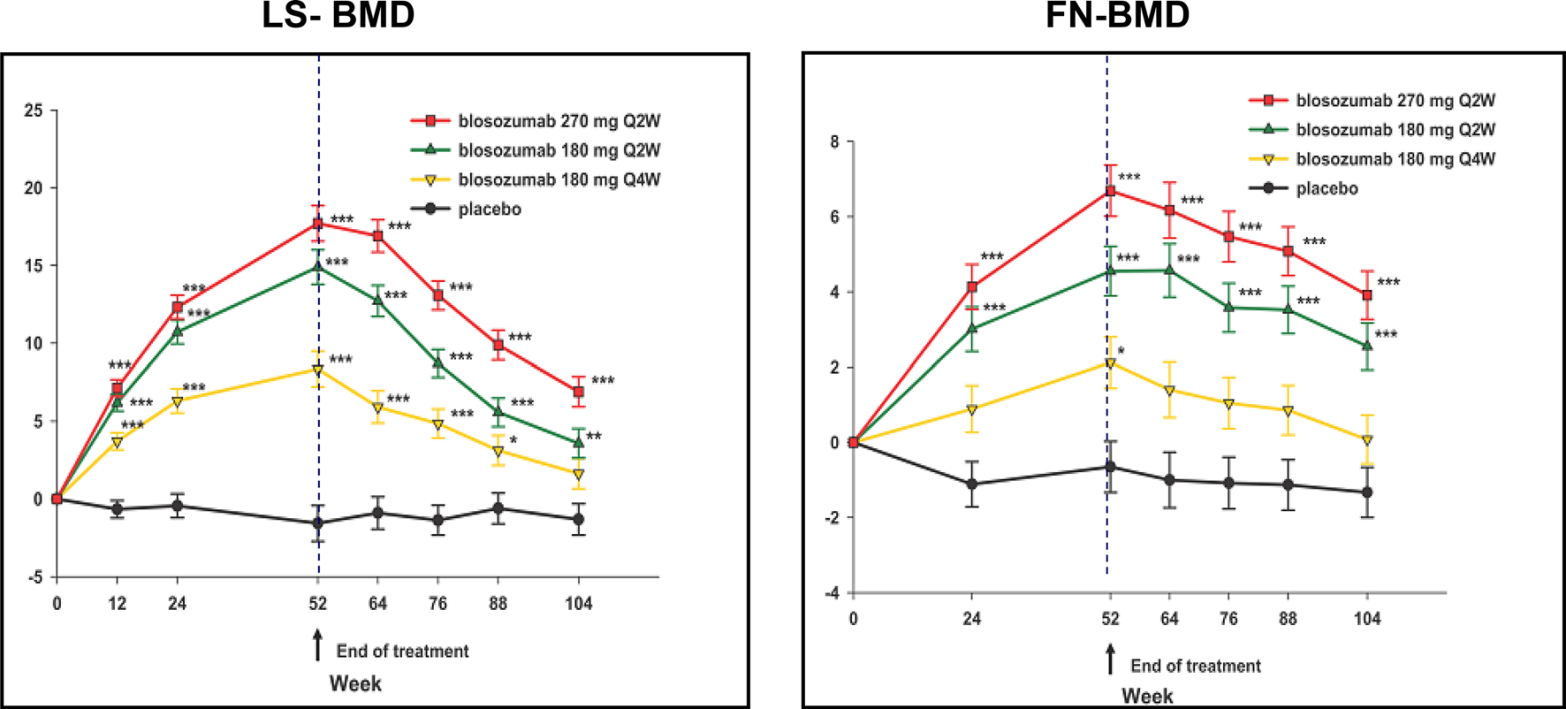
Percent changes of lumbar spine and femoral neck BMD during and after treatment of postmenopausal women with low bone mass with 3 different doses of blosozumab. (From Ref. [63]). ***p < 0.001, **p < 0.01, *p < 0.05

The results of a dose-finding study of blosozumab were recently reported [62]. One hundred and twenty postmenopausal women aged 45–85 years with BMD T-scores between −2.0 and −3.5 were included in the study and 106 completed 1 year of treatment. All women received calcium and vitamin D supplements and were randomized to receive placebo or blosozumab subcutaneously 180 mg every 4 weeks, 180 mg every 2 weeks, or 270 mg every 2 weeks. Blosozumab treatment induced dose-dependent increases in lumbar spine BMD of 8.4, 14.9, and 17.0 %, in total hip BMD of 2.1, 4.5, and 6.3 %, and in femoral neck BMD by 2.7, 3.9, and 6.3 %, respectively. Total body bone mineral content increased also dose-dependently after 1 year; blosozumab treatment had no effect on BMD of the distal radius. Mild injection site reactions were more frequently observed with blosozumab than with placebo and antibodies to blosozumab developed in 32 patients (35 %) in one of whom these antibodies were neutralizing and reduced her response to treatment. Although the frequency of adverse events was similar among all groups, four women (all Japanese) treated with blosozumab were diagnosed with breast cancer between 3 months after start of treatment to 1 year after the last dose of the antibody while no cases of breast cancer were reported in the placebo-treated women. These were not considered to be related to treatment. To evaluate the effect of discontinuation of blosozumab on BMD and bone turnover markers, the 106 women who completed the first year of the study were followed for an additional year without treatment; eighty-eight women completed the study [63]. Following discontinuation of blosozumab, spine and hip BMD decreased progressively with similar rates for all doses reaching values that depended on the peak values achieved on treatment after 1 year (Fig. 6). For example, in women treated with the highest dose of blosozumab BMD values were still significantly higher than baseline values. Conversely, in women treated with the lowest dose of blosozumab BMD values returned to baseline after 1 year. Bone turnover markers showed no particular changes and remained around baseline values with small but significant increases in serum CTX with some but not all doses of blosozumab. There is no information about on-going phase III clinical studies with blosozumab (www.clinicaltrials.gov).

No results have been communicated yet with the use of BPS804 but a randomized, double-blind, placebo-controlled, phase II study evaluating the safety and efficacy of multiple-dosing regimens of BPS804 in postmenopausal women aged 45–85 years with baseline lumbar spine T-score between −2.0 to −3.5 was completed in 2015 (www.clinicaltrials.gov).

### Clinical Perspective

The results of the preclinical and early clinical studies with sclerostin inhibitors demonstrate a new treatment paradigm with a bone building agent for the management of patients with osteoporosis, the antifracture efficacy, and long-term tolerability of which remain to be established in the on-going phase III clinical studies. The findings in animals and humans with sclerostin deficiency as well as the documented kinetics of bone remodeling/modeling and bone mass in response to sclerostin inhibitors though clearly indicative of a novel mechanism of action, do not yet allow an accurate estimate of the optimal duration of treatment. Osteoporosis is a chronic disease requiring chronic treatment and it is not yet known whether long-term treatment with a sclerostin inhibitor will be associated with a sustained anabolic effect on bone or whether initial treatment need to be followed by another agent. The latter approach seems more likely and currently available data suggest that this may be preferred for the pharmacological management of patients with severe osteoporosis in the future.
